# Influence of different electrode belt positions on electrical impedance tomography imaging of regional ventilation: a prospective observational study

**DOI:** 10.1186/s13054-015-1161-9

**Published:** 2016-01-08

**Authors:** Jan Karsten, Thomas Stueber, Nicolas Voigt, Eckhard Teschner, Hermann Heinze

**Affiliations:** 1 Department of Anaesthesiology and Intensive Care Medicine, Hannover Medical School, Carl-Neuberg-Str. 1, 30625, Hannover, Germany; 2 Draeger Medical GmbH, Moislinger Allee 53, 23558 Lübeck, Germany; 3 Department of Anaesthesiology and Intensive Care Medicine, University of Lübeck, Ratzeburger Allee 160, 23538 Lübeck, Germany

## Abstract

**Background:**

Electrical impedance tomography (EIT) is a non-invasive bedside tool which allows an individualized ventilator strategy by monitoring tidal ventilation and lung aeration. EIT can be performed at different cranio-caudal thoracic levels, but data are missing about the optimal belt position. The main goal of this prospective observational study was to evaluate the impact of different electrode layers on tidal impedance variation in relation to global volume changes in order to propose a proper belt position for EIT measurements.

**Methods:**

EIT measurements were performed in 15 mechanically ventilated intensive care patients with the electrode belt at different thoracic layers (L1-L7). All respiratory and hemodynamic parameters were recorded. Blood gas analyses were obtained once at the beginning of EIT examination. Off-line tidal impedance variation/tidal volume (TV/VT) ratio was calculated, and specific patterns of impedance distribution due to automatic and user-defined adjustment of the colour scale for EIT images were identified.

**Results:**

TV/VT ratio is the highest at L1. It decreases in caudal direction. At L5, the decrease of TV/VT ratio is significant. We could identify patterns of diaphragmatic interference with ventilation-related impedance changes, which owing to the automatically adjusted colour scales are not obvious in the regularly displayed EIT images.

**Conclusions:**

The clinical usability and plausibility of EIT measurements depend on proper belt position, proper impedance visualisation, correct analysis and data interpretation. When EIT is used to estimate global parameters like VT or changes in end-expiratory lung volume, the best electrode plane is between the 4th and 5th intercostal space. The specific colour coding occasionally suppresses user-relevant information, and manual rescaling of images is necessary to visualise this information.

## Background

Pulmonary complications, either postoperative or during the course of a critical illness, have major influence on patient outcome [1, 2]. Accumulating evidence suggests that quality of mechanical ventilation can attenuate, prevent, or worsen those pulmonary complications [3, 4]. Nevertheless, it is unclear how exactly a “lung protective approach” should be performed, which patients may benefit and whether “one size fits all” approaches meet the requirements for protective mechanical ventilation. From a physiological point of view, an individualised approach which adapts tidal volume (VT) and positive end-expiratory pressure (PEEP) to a patient’s specific lung aeration and mechanics seems reasonable. Electrical impedance tomography (EIT) is a non-invasive bedside tool which allows an individualised ventilator strategy by monitoring tidal ventilation and lung aeration [5, 6]. It generates cross-sectional images of the distribution of impedance changes within the chest for monitoring the distribution of regional ventilation [7–9]. This technique is increasingly used in clinical anaesthesia and critical care. However, clinical studies proving benefit on patient’s outcome are missing, although EIT-tailored ventilation showed benefits in experimental data and on human surrogate endpoints [10–13]. For visualisation of regional ventilation distribution, a 4-cm broad electrode belt is applied around the chest, which measures impedance changes in a lens-shaped slice of the chest. Impedance changes of this lens-shaped intra-thoracic volume contribute to the generation of EIT images. The volume is defined as EIT sensitivity region. Its thickness increases toward the central region of the body (up to a thickness of approximately 12 cm). Electrical impedance changes due to tidal change of lung volume are assessed by the EIT, and thus overdistension and recruitment of regional lung areas can be estimated. EIT can be performed at different cranio-caudal thoracic levels, but data about the optimal belt position are missing. Furthermore, automatic adjustment of the colour scale for EIT images (vertical axis limit) may impair recognition of impedance changes due to effects other than ventilation.

This study is a prospective observational study. The main goal was to evaluate the impact of different electrode layers on the tidal impedance variation/tidal volume (TV/VT) ratio and certain patterns of impedance distribution. We hypothesise that TV/VT ratio is capable of describing phenomena concerning EIT at different electrode levels. Thereby, we especially focused on patterns of impedance distribution in caudal, juxta-diaphragmatic electrode levels.

## Methods

The study protocol was approved by the local ethics committee of the Hannover Medical School (chair: Hans-Dieter Tröger) (#2240-2014), and written informed consent was obtained from all patients or their legal representatives. We included 16 ventilated patients in our interdisciplinary intensive care unit (Department of Anaesthesiology and Intensive Care Medicine, Hannover Medical School, Germany). There were no exclusion criteria for participation except thoracic deformation, active implants and skin lesions. During the observation, technical problems led to exclusion of one patient from off-line analysis. Thus, 16 patients were assessed for eligibility, but 15 patients were allocated passing through the study protocol and were analyzed.

### EIT

Measurements were obtained by using the PulmoVista 500 (Draeger Medical GmbH, Lübeck, Germany). A silicone belt with 16 equidistant electrodes was placed around the patient’s chest at the level to be imaged. Data were generated by applying low constant-amplitude alternating electrical currents (108 kHz, 9.1 mA) to an adjacent pair of electrodes. Resulting voltage differences between adjacent electrode pairs were measured in a sequential rotating process (frame rate 20 Hz). A low-pass filter was used to eliminate cardiac oscillations.

The EIT images were generated by using a modified Newton–Raphson reconstruction algorithm (Draeger EIT Data Analysis Tool 6.1; Draeger Medical GmbH). After image reconstruction, the relative impedance changes are translated into a colour scale. Impedance changes of less than 10 % of the determined maximum impedance change are coloured black. Impedance changes above 10 % of the maximum impedance change are displayed in dark blue/light blue and white. Regions with minor negative impedance changes are inhibited and coloured black in status images (which are used in our study). The colour scale is adjusted continuously (“auto-scaled”) to display the dynamics of regional ventilation independently of VT changes or influences on bioelectric properties. The zero position of the colour scale is always located in a position which provides 85 % for the display of positive changes, while negative impedance changes are compressed to 15 % of the colour scale. By this, the negative values of impedance changes are weighted differently than the positive ones. Auto-scaling leads to a display of mainly positive impedance changes and is specific for PulmoVista data visualisation [14, 15].

### Study protocol

The EIT measurements were performed in a supine position with the upper part of the body elevated (30°). Patients were ventilated according to the attending physicians’ respirator adjustments (EVITA 4; Draeger Medical GmbH). Patients were ventilated in pressure-controlled mode (biphasic intermittent positive airway pressure). The silicone EIT belt was placed around the thoracic circumference at the highest possible level (below the armpits) at the (third)/fourth intercostal space (ICS). Measurements were always performed by two investigators to ensure an orthogonal plane of electrodes. Furthermore, we used the specific ruler (provided by the manufacturer to choose the correct belt size) for guidance. The study-related definition of EIT sensitivity regions is based on layers (L1, L2, etc.). The first belt position was labelled layer 1 (L1). The next belt position (L2) was defined 2 cm caudal L1 (half width of EIT belt). L3–L7 were defined similarly (L3: 2 cm caudal L2; L4: 2 cm caudal L3; L5: 2 cm caudal L4; L6: 2 cm caudal L5; L7: 2 cm caudal L6). According to these layers, we provide the corresponding ICS in terms of anatomical attribution, where EIT measurements were performed: (1) L represents the cranio-caudal shift of the electrode belt. (2) ICS provides anatomical attribution. Stating centimeters for the range where TV/VT ratio does or does not change significantly can be useful for clinical users. In all patients, we examined the juxta-diaphragmatic level. The number of potential layers depends on, among other things, the body’s structure, heights and physiognomy as well as pathophysiological circumstances (i.e., elevated diaphragm, increased intra-abdominal pressure, obesity). Considering the rules of our local ethics committee, we stopped examinations when certain patterns of impedance distribution occurred, which are described in detail in the Results and Discussion sections.

Measurements started after a stabilisation period of 5–10 minutes. Before each measurement, a signal check was performed in accordance with the recommendations of the manufacturer (skin-electrode resistance, signal-to-noise ratio). EIT measurements lasted more than 2 minutes per layer. During the measurements, the EIT device was connected with the respirator via medibus interface, and all respiratory parameters, including airway pressures (peak inspiratory pressure and PEEP), VT, respiratory rate and dynamic compliance of the respiratory system (C_res_), were directly stored online. Hemodynamic parameters were assessed by a standard perioperative monitoring device (CARESCAPE B650; GE Healthcare GmbH, Munich, Germany).

### Off-line analysis

The EIT data were stored by the device and analysed off-line. EIT raw data (i.e., functional status images, TV, impedance curves and numerical values) were used for data processing. The baseline was repeatedly defined at each examination level. TV is defined as the difference of end-inspiratory relative impedance change (∆Z) and end-expiratory ∆Z. Numeric values are continuously calculated and displayed over time to quantify regional impedance changes and to express the ventilation distribution as regional proportions. Images are a 32 × 32 pixel colour-coded matrix (white: positive/highest ∆Z; blue: medium ∆Z; black: impedance changes less than 10 %; purple: negative ∆Z, caused by inverted signals in the affected region). Global impedance curves primarily display impedance changes related to ventilation over time. There is usually a strong correlation between this curve and the volume curve displayed by the ventilator. Regional impedance curves display the sum of impedance changes within the specified region-of-interest (ROI) over time. For proof of cranio-caudal impedance changes, the TV/VT ratio was calculated (averaged over 1 minute). For regional TV/VT ratio, the EIT was equally divided up into two horizontal non-overlapping ROIs (32 × 16): dependent/dorsal and non-dependent/ventral ROI. In addition, the proportion of negative impedance changes in cranio-caudal direction was quantified. The area of negative impedance changes was calculated from EIT status images and divided by the area of the whole 32 × 32 pixel image (GSA Image analyser version 4.0.3.; GSA Gesellschaft für Softwareentwicklung und Analytik mbH, Rostock, Germany). Chest x-ray, which was performed according to the intensivist in charge, provided a basis for anatomical orientation and visualised any specific anatomical circumstances (e.g., diaphragmatic elevation, heart outline).

### Statistics

Quantitative variables were described as mean and standard deviation unless stated otherwise. Qualitative variables were expressed as proportions and association between these variables. Statistical analysis was performed by using GraphPad Prism 5 (GraphPad Software, Inc., San Diego, CA, USA). After Kolmogorov-Smirnov test and owing to small sample size, statistical differences between quantitative variables were determined by using one-way analysis of variance (non-parametric Kruskal-Wallis test) with Dunn’s *post hoc* testing for pairwise multiple comparisons. Significance was considered as a *P* value of less than 0.05.

## Results

Baseline parameters presenting the physiologic and demographic data of the study population are given in Table 1. Patients had a predicted mortality of more than about 10–20 % based on the Simplified Acute Physiology Score II (SAPS II) at the time of the study (SAPS II 35 ± 10.6). According to the body mass index, the study population can be classified pre-obese to obesity grade I. Regarding oxygenation, a moderate to mild impairment of oxygenation was seen in most patients. There were no significant differences in the ventilator settings during the measurements at the electrode levels (Table 2). Patients were ventilated with a VT of 8.2 ± 1.8 ml VT as predicted by body weight.

**Table 1:** Baseline characteristics of patients (n = 15)

Age, years	62.6 ± 14.8
Females/Males	1/14
Smoker/Non-smoker, %	38.5/61.5
SAPS II	35 ± 10.6
Body weight, kg	91.1 ± 10.3
Height, cm	178.5 ± 7.2
BMI, kg/m^2^	28.8 ± 4.4
Heart rate, 1/min	75 ± 16
MAP, mm Hg	81 ± 11
Catecholamines, yes/no	4/11
PaO_2_, mm Hg	114 ± 42
PaCO_2_, mm Hg	44 ± 8
SaO_2_, %	97 ± 2
PF ratio, mm Hg	255 ± 75

*SAPS II* Simplified Acute Physiology Score II, *BMI* body mass index, *MAP* mean arterial pressure, *PaO*_*2*_ arterial oxygen partial pressure, *PaCO*_*2*_ arterial carbon dioxide partial pressure, *SaO*_*2*_ arterial oxygen saturation, *PF ratio* ratio of arterial oxygen partial pressure to fractional inspired oxygen. Data are mean ± standard deviation unless indicated otherwise

**Table 2:** Respiratory parameters

	L1	L2	L3	L4	L5	L6	L7	*P* value
RR, 1/min	15.7 ± 3.6	15.5 ± 3.6	15.8 ± 3.8	15.7 ± 4.0	15.6 ± 3.7	15.9 ± 3.7	16.5 ± 3.9	n.s.
VT, ml	649 ± 134	637 ± 141	627 ± 138	621 ± 132	611 ± 125	639 ± 154	607 ± 122	n.s.
VT PBW, ml/kg	8.6 ± 1.8	8.3 ± 1.8	8.2 ± 1.9	8.1 ± 1.8	8.1 ± 1.8	8.3 ± 2.1	7.7 ± 1.5	n.s.
PEEP, mbar	8.0 ± 2.4	8.1 ± 2.3	8.1 ± 2.3	8.1 ± 2.3	8.1 ± 2.3	8.1 ± 2.5	7.8 ± 2.3	n.s.
PIP, mbar	20.7 ± 4.7	20.3 ± 4.7	20.2 ± 4.6	20.2 ± 4.6	20.2 ± 4.5	20.2 ± 4.8	18.5 ± 4.2	n.s.
C_res_, ml/mbar	83.3 ± 43.3	81.1 ± 35.7	74.7 ± 25.7	74.8 ± 26.7	75.9 ± 28.1	78.4 ± 30.4	83.2 ± 35.9	n.s.

*RR* respiratory rate, *n.s.* not significant, *VT* tidal volume, *PBW* predicted body weight (males: PBW = 50 + 0.91 x (height in cm – 152.4), females: PBW = 45.5 + 0.91 x (height in cm – 152.4), *PEEP* positive end-expiratory pressure, *PIP* peak inspiratory pressure, *C*_*res*_ respiratory compliance. Data are mean ± standard deviation unless indicated otherwise. L1-L5: n = 15, L6: n = 12; L7: n = 8

**Fig. 1:**
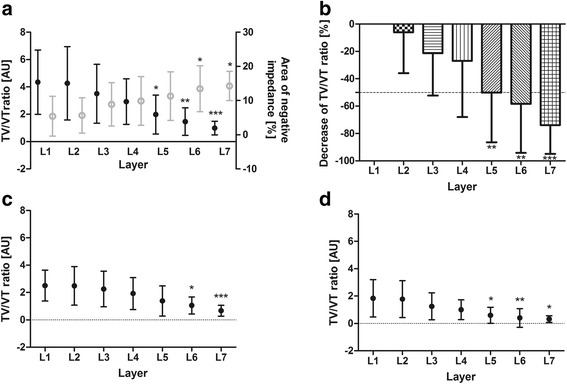
TV/VT ratio at different layers. Interrelation of global TV/VT ratio (*black*) and amount of negative impedances (*grey*) (**a**). Percentage decrease of TV/VT ratio (relative) deeming L1 as baseline (**b**). Regional TV/VT ratio (**c, d**) at different layers from cranial (L1) to caudal (L7). **c** Non-dependent/ventral. **d** Dependent/dorsal. Data are mean ± standard deviation. Kruskal-Wallis test with Dunn’s *post hoc* testing for pairwise multiple comparison. Significance compared with L1: *P < 0.05, **P < 0.01, ***P < 0.001. *AU* arbitrary units, *L* layer, *TV/VT* tidal impedance variation/tidal volume ratio

TV/VT ratio shows a significant decrease from L5 (*P* < 0.0001), which is more than about 10 cm below the most cranial electrode level (L1, i.e., intercostal space (3-)4 in parasternal line) (Fig. 1a). Figure 1c and d show the course of regional TV/VT ratio (non-dependent [c] and dependent [d] lung regions). There was a significant decrease of TV/VT ratio in L5, L6 and L7 (*P* = 0.0002) in the dependent lung regions, whereas in non-dependent lung regions, a significant decrease of regional TV/VT ratio occurred later (L6, L7) (*P* < 0.0001). Given the percentage loss of TV/VT (Fig. 1b), a mean of more than 50 % of baseline TV/VT ratio (L1) can be considered statistically significant (*P* < 0.01). The area of negative impedance changes increases in cranio-caudal direction (*P* < 0.002) (Fig. 1a).

**Fig. 2:**
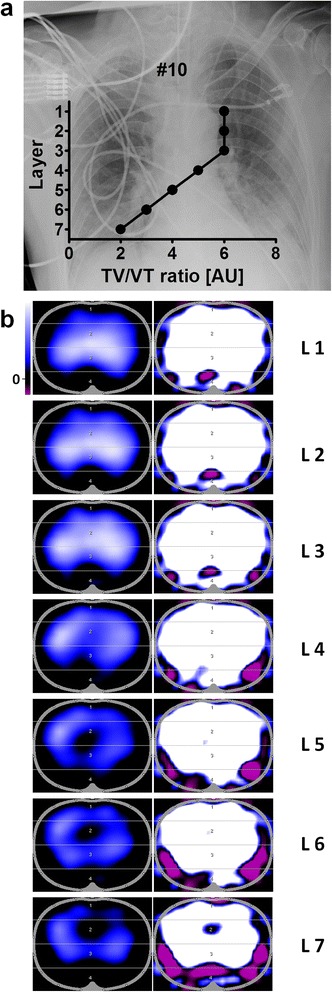
Influence of belt position on electrical impedance tomography in patient 10. **a** TV/VT ratio depends on belt position. The ratio decreased in cranio-caudal direction (L1/ICS 4; L7/ICS 8). **b** Minute images of different layers in cranio-caudal direction were displayed as examples. Functional status images with automatically adjusted colour scales (assigning white to the maximum impedance change) are displayed on the left side. On the right side, colour scales were unadjusted to visualize interference. Images are a 32 × 32 pixel coloured matrix (white: positive/highest ∆Z; blue: medium ∆Z; black: impedance changes less than 10 %; purple: negative ∆Z, inverted signal). *ICS* intercostal space, *L* layer, *TV/VT* tidal impedance variation/tidal volume ratio

**Fig. 3:**
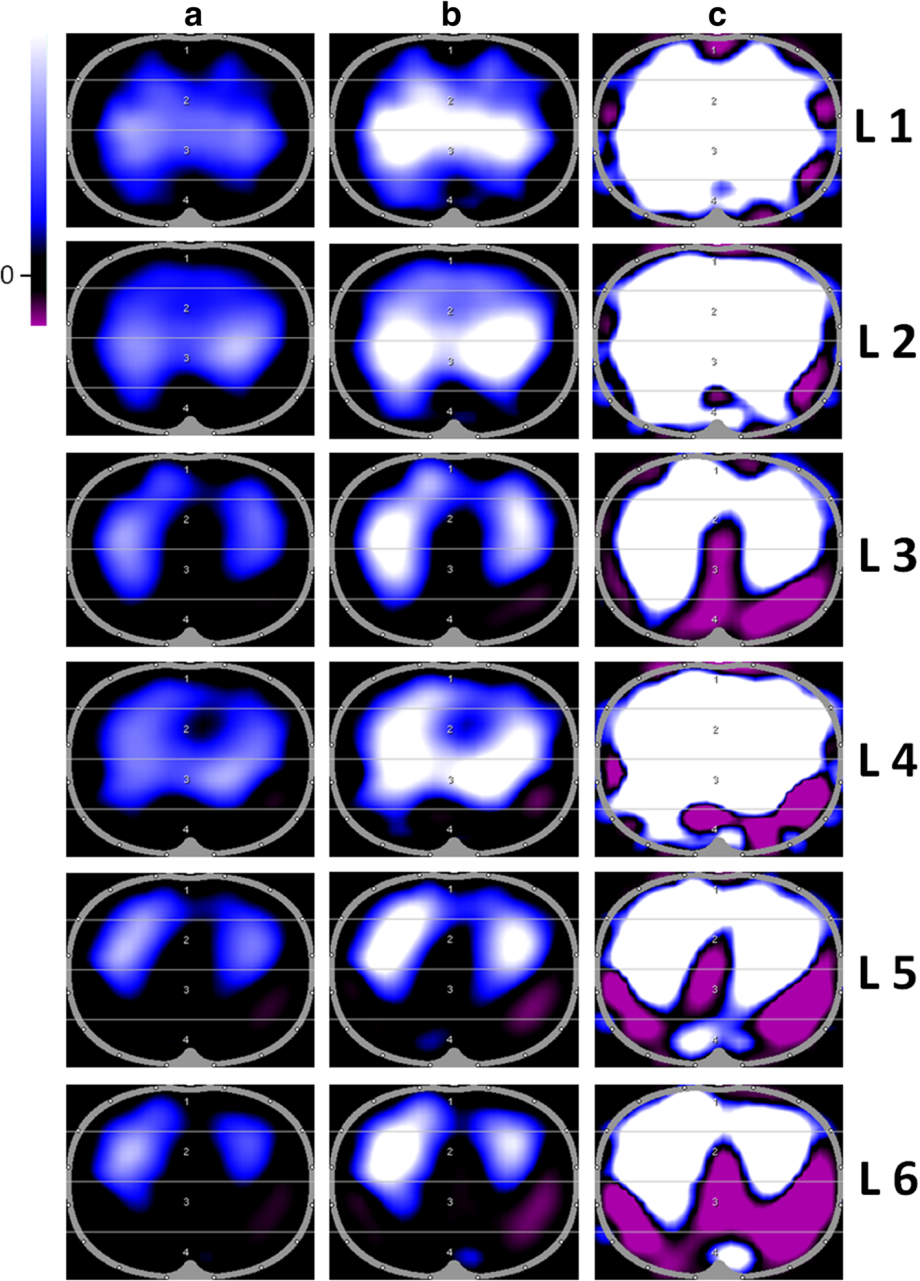
Influence of colour scales on functional electrical impedance tomography images. Examples of minute images in cranio-caudal direction (L1 to L6) (patient 1) with different colour scale endpoints: (**a**) automatically adjusted colour scales (assigning white to the maximum impedance change), (**b**, **c**) user-defined adjusted colour scales. Massive pleural effusion could be excluded. Images are 32 × 32 pixel coloured matrix (white: positive/highest ∆Z; blue: medium ∆Z; black: no impedance change; purple: negative ∆Z, inverted signal). *L* layer

**Fig. 4:**
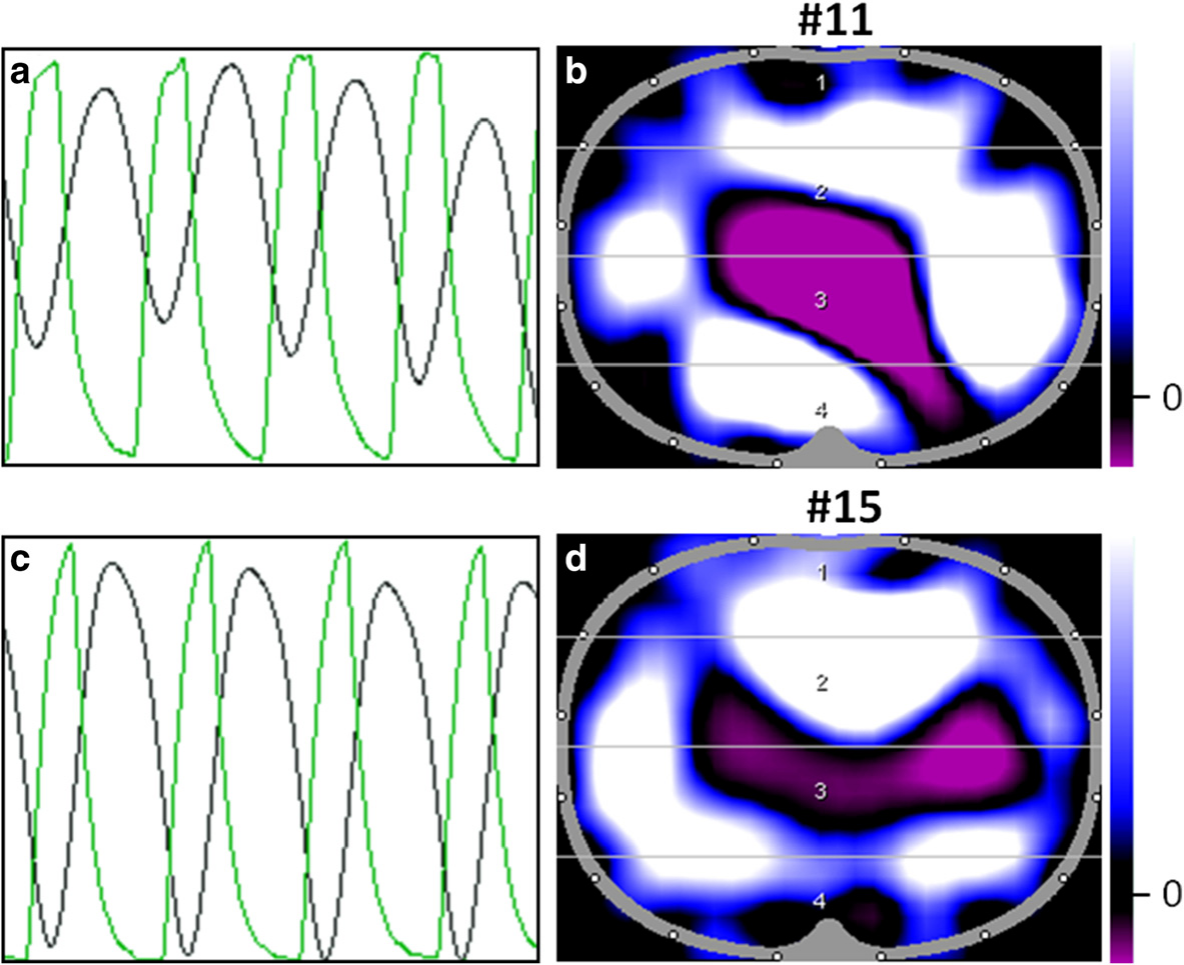
Out-of-phase of impedance and tidal volume signal. **a**, **c** Impedance curves are painted black, and tidal volume curves are painted green. **b**, **d** Corresponding functional status images: the global impedance signals (blue/white) are induced mainly by the diaphragm (patient 11 L5, 12 cm below the armpits; patient 15 L7, 16 cm below the armpits)

Representative examples of functional EIT images (Figs. 2 and 3) show typical patterns of impedance distribution at different thoracic layers. Figure 2 is an example of the course of TV/VT ratio at different thoracic layers in cranio-caudal direction. Figure 3 shows the impact of colour scales in functional EIT images on the visualisation of impedance distribution. Figure 4 shows representative examples of phase opposition of impedance and volume curves (Fig. 4).

## Discussion

In this study, we showed the influence of different EIT belt positions in 15 critically ill patients. The correlation between VTs measured by a synchronized mechanical ventilator and the global changes in impedance (sum of pixel values) strongly depends on the electrode belt levels where the EIT measurements were performed. This holds true for the whole cross-sectional area of the thorax (representing a lens-shaped volume of the chest) as well as for the non-dependent/anterior or dependent/posterior half of the respective cross-sectional area.

We also show that the PulmoVista-specific colour coding occasionally suppresses user-relevant information and that manual rescaling of images is necessary to visualise this information (i.e., phase-inverted signals at juxta-diaphragmatic levels). These findings are relevant for future direction of research and clinical application.

### TV/VT ratio

The correlation between VTs and TV changes with regard to the position of the belt. We observed the highest correlation of impedance changes (sum of pixel values) and VT (from ventilator) when the electrode belt was applied between ICS (3)/4 and ICS 5. The correlation decreased progressively to less than 50 % at distances 10–14 cm below this plane. Measurements carried out in the most caudal electrode levels are certainly correct but more difficult to analyse and likely to be misinterpreted. This is especially relevant during PEEP trials where PEEP-induced changes of the diaphragmatic position may alter the TV/VT ratio, which could then be mistaken as changes of VTs. In contrast, the impact of PEEP-induced changes of the diaphragmatic position on the TV/VT ratio is probably minor when measurements are conducted at levels where the ratio does typically not change significantly. Furthermore, TV/VT ratio is an individual measure. Absolute values are initially not suited to carry out inter-subject comparisons but to assess the variability of findings.

It has to be taken into account that EIT determines regional VTs which cannot be expected to be identical all over the lungs. Consequently, TV/VT ratio cannot be expected to be the same in all examined EIT sensitivity regions, because the relative amount of lung tissue in the cross-sectional plane changes depending on the cranio-caudal location. However, at more caudal thoracic levels (≥L5) where the TV/VT ratio decreased significantly, the mediastinal cavity and the diaphragm entering the electrode plane seem to contribute most to this decrease. Interestingly, significant decrease of regional TV/VT ratio in dependent dorsal lung regions occurs earlier than in non-dependent ventral lung regions. We hypothesise that this difference is most likely due to the development of basal atelectasis formation in supine position and thus leads to a cranial shift of the diaphragm in dorsal-dependent lung regions [16, 17]. Nevertheless, we cannot provide proof of this phenomenon, because we did not perform computed tomography (CT) and this can be seen as a limitation of the study.

### Impact of Newton–Raphson algorithm and automatically adjusted and user-defined colour scales

**Fig. 5:**
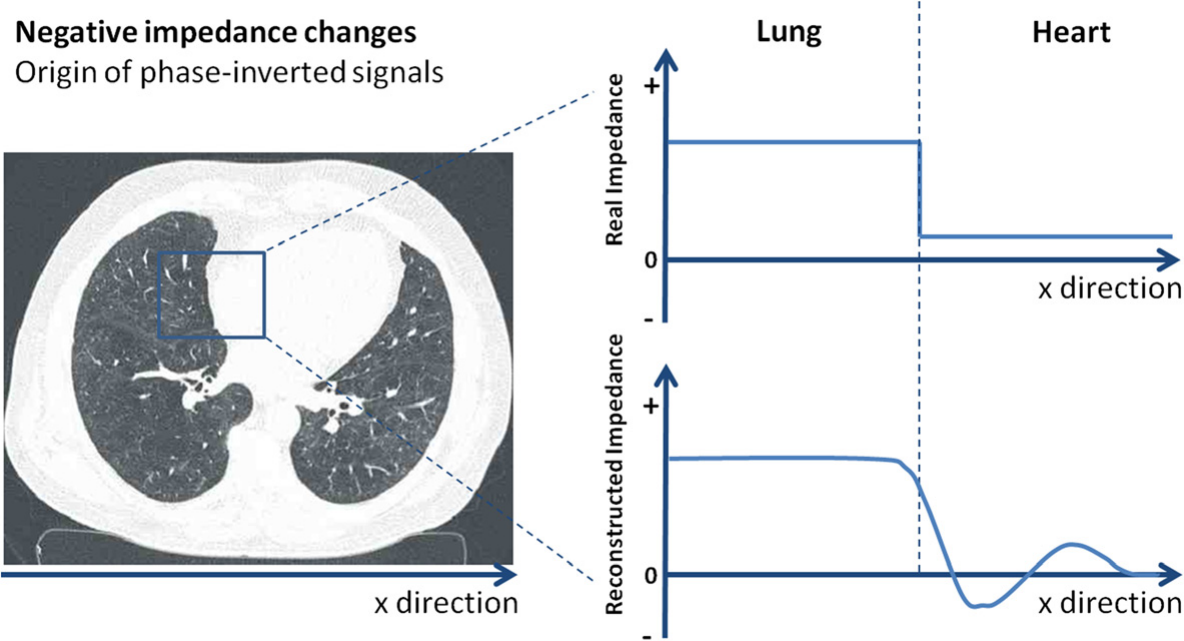
Negative impedance changes. Origin of phase-inverted signals in reconstructed electrical impedance tomography images (representative example)

There are characteristics of both the genuine reconstruction algorithm (i.e., Newton–Raphson) and the specific EIT data visualisation algorithm, which impact our findings. In the presence of certain physiological conditions, the reconstruction algorithm may introduce overshoot effects leading to areas of large negative impedance changes, which are displayed in purple. This effect may occur when regions of high conductivity (e.g., the diaphragm/mediastinum, pleural effusion) and low conductivity (e.g., well-ventilated lung region) are located next to each other (Fig. 5). The overshoot phenomenon may cause inverted regional impedance changes relative to the global impedance waveform. This results in negative values of the corresponding parameters for the regional status image. By this, especially at tissue boundaries, the algorithm can induce artifacts which have to be interpreted correctly [14, 15].

Another implication of our study on EIT data visualisation is a careful consideration of the definition of the colour scale in functional EIT images (Fig. 3). Automatically adjusted colour scales may mask these mentioned effects, but the endpoint of the colour scale is just a variable of data visualisation at the user interface. EIT raw data providing the basis for all calculations are unaffected. A user-defined adjusted colour scale (Fig. 3b and c) unmasks the appearance of impedance changes with negative amplitude (i.e., mediastinal cavity and diaphragmatic movement). This has to be taken into account when interpreting EIT data by functional EIT images at bedside. The observed effect of the colour scale is specific for the PulmoVista device we used in our study [14]. Although auto-scaling provides an optimised continuous display of the ventilation distribution, it prevents these images from being used for the analysis of phase-inverted signals at juxta-diaphragmatic levels [14, 15].

### EIT sensitivity region in previous studies

The validity of EIT has been proven several times, but different results have been found comparing (regional) EIT parameters and global lung parameters such as end-expiratory lung volume (EELV). We did not perform any EELV measurements, but we want to provide a succinct explanation of the implications of our findings, particularly in relation to previous related studies. Therefore, we need to refer to the results of van Genderingen et al. [18], Hinz et al. [19] and Bikker et al. [20], although the results of the last of these were in concordance with ours. A linear relationship between impedance change and volume change has been previously established by van Genderingen et al., for example. Hinz et al. showed a linear correlation between lung volume by EIT and by an open-circuit nitrogen washout manoeuvre, whereas Bikker et al. [20] denied these findings, showing that TV did not equal VT at one thoracic level. In a previous study, we found that EELV cannot be estimated by EIT with reasonable accuracy [21]. In our opinion, one of the key confounding factors in those previous studies may have been the choice of the electrode plane because EIT can be performed at different thoracic levels. If the portion of lung tissue which is captured within the EIT sensitivity region is altered (particular interference at juxta-diaphragmatic level but also influence of PEEP-induced cranio-caudal shift of intrathoracic structures), a lower correlation of volume changes within the EIT sensitivity region and EELV changes is comprehensible.

EIT measurements at a juxta-diaphragmatic level implicate a potential shift of morphologic structures into the EIT sensitivity region affecting data interpretation. A position close to the diaphragm may lead to misinterpreted measurements. On the other hand, a position too apical near the apex of the lung may miss visualisation of ventilation in the dependent and probably the most vulnerable parts of the lung which would benefit most from individualised ventilator settings. Most previous studies have been performed attaching the electrode belt around the sixth intercostal space. Physiological changes (e.g., increased intra-abdominal pressure) or changes in ventilator settings (e.g., PEEP trials) lead to caudo-cranial shifts of intrathoracic structures and affect patterns of impedance distribution. This is supported by a further study from Bikker et al., who showed that PEEP changes can alter the portion of lung tissue visualised by the EIT [22].

### EIT sensitivity region at a juxta-diaphragmatic level

In our study, we give examples of the diaphragm entering the EIT sensitivity region at lower layers (≥L5) which affect computed TV/VT ratio (Figs. 2, 3 and 4). These effects may be aggravated by PEEP variation or other physiologic changes in clinical practice (e.g., patients with acute respiratory failure and formation of atelectasis in dependent lung regions and potential diaphragmatic elevation). Bikker et al. [22] tried to avoid these effects by choosing a small ROI away from the middle of the EIT image where – from their point of view – the effects of the diaphragm are most likely. In our opinion, this approach may be misleading for two reasons. First, a smaller ROI means an additional loss of spatial resolution anyway. Theoretically, the EIT sensitivity region is 4 cm thick at the thoracic surface (electrode belt) and thickness increases toward the thoracic centre. The mapping of complex three-dimensional morphological structures and its bioelectric properties on a two-dimensional template further decreases spatial resolution. Hence, the displayed thoracic layer does not necessarily equal the corresponding anatomic layer, and the visualised impedance changes do not match precisely with their localization within the thorax. Secondly, morphologic structures both in the middle and in peripheral (dependent) regions of EIT images (i.e., the mediastinal cavity and the diaphragm) may potentially lead to EIT findings (Fig. 3), which are difficult to analyse and interpret. Figure 4, for example, shows out-of-phase signals of impedance and volume curves. Occurence of the out-of-phase signal in lower examination planes means that impedance curves and VT curves are not congruent: relative impedance maxima (black curve) can be detected in expiration (green curve) and vice versa. This phenomenon is caused by reconstruction artifacts (Fig. 5) and specific software algorithms detecting (impedance-based) begin of inspiration and expiration (breath detection algorithm). In Fig. 4, the global impedance signal with positive amplitude (in the EIT images represented by blue or white colour) is probably induced by diaphragmatic movement. The provided EIT tool uses an automatic breath detection, which assumes that the maxima of the global ∆Z curve always represent the end of inspiration. However, if the belt is placed in the juxta-diaphragmatic position, the impedance changes caused by diaphragmatic movement may be larger than the ventilation-related impedance changes. In this condition, the breath detection algorithm misinterprets the diaphragmatic impedance changes as ventilation-related impedance changes. Consequently, the global ∆Z curve (black) and the volume curve as transmitted by the ventilator (green) show out-of-phase-signals (Fig. 4). On the contrary phase lags in regional impedance curves and changes in curve shape may occur if atelectatic lung regions are affected by either delayed filling (different time constant) [23] or tidal recruitment. In regard to this matter, three different modalities of asynchrony from ventilation signal can be described: (1) inspiratory and expiratory phase lag between regional impedance (ventilation) and global impedance curves due to different regional ventilator time constants (regional delay), (2) inspiratory phase lag but premature expiration of the regional impedance curve due to tidal recruitment, and (3) out-of-phase signals of the global impedance and volume curves due to the diaphragm entering the EIT sensitivity region with every breath [14, 15].

Considering the usage of more than one belt is technically difficult because of hardware and software problems and probably does not lead to additional information. Reske et al. found that one single juxta-diaphragmatic CT slice reflects the status of atelectatic-dependent lung regions better than a combination of CT slices representing the whole lung. Volume changes measured in one thoracic plane (i.e., ICS 5) were found to be representative for the whole lung [24]. Probably, these experiences can be applied to EIT measurements.

### Clinical considerations

#### Spontaneous breathing

Finally, we suppose that our findings apply only partly to spontaneous breathing. First, in supine spontaneously breathing patients, dorsal-dependent lung regions are preferentially ventilated and this affects regional TV/VT ratio. Secondly, chest geometry changes and diaphragm configuration are altered in mechanically ventilated patients. By this, one can assume that characteristics of the EIT sensitivity region, respectively patterns of impedance distribution and diaphragmatic interference at most caudal electrode levels, are affected in a different way. Krueger-Ziolek et al. showed that electrode positioning systematically influences EIT imaging in spontaneously breathing patients [25]. Nevertheless, the authors suggest applying the electrode belt in a mid-thoracic plane (i.e., ICS 5), which is in concordance with our findings.

#### Gender

Generally, the effects described in our study can be seen in both men and women. Systematic EIT measurements in women revealed the following: in women, we suggest applying the belt slightly below the breast because of breast anatomy. However, in any cases, it is necessary to evaluate the patterns of impedance distribution because in almost 50 % of evaluated women there were lateral overshoots; that is, negative impedance changes occur (unpublished data). These findings coincided with the results of a previous published study by Krueger-Ziolek et al. [25].

## Conclusions

We examined 15 mechanically ventilated patients by EIT with the electrode belt placed in seven cranio-caudally shifted locations. Until today, there exist published data on only two or three electrode planes. The present study confirms the previous results showing the dissimilar ventilation distribution patterns among the planes. We also show that the PulmoVista-specific colour coding occasionally suppresses user-relevant information and that manual rescaling of images is necessary to visualise this information (i.e., phase-inverted signals at juxta-diaphragmatic levels). TV/VT ratio is capable of describing phenomena concerning EIT at different electrode levels. The TV/VT ratio decreases in cranio-caudal direction, whereas the proportion of negative impedance changes increases in cranio-caudal direction. TV/VT ratio did not change significantly in the range of less than 10 cm (below the armpits). The course of TV/VT ratio and tentative changes of colour scales may be a practical approach to assess the accuracy of EIT data at the level to be imaged. In the presence of (lateral) negative impedance changes, it should be examined whether this may be due to diaphragmatic movement. We suggest applying the electrode belt at ICS 4–5 (parasternal line). In view of our results, we think there should be a consensus on a standard electrode plane for pulmonary EIT monitoring, specifically regarding a standard approach to evaluate the correct placement of the electrode belt in future clinical and experimental applications.

## Key messages

- The clinical usability and plausibility of EIT measurements depend on proper belt positioning, proper impedance visualisation, correct analysis and data interpretation.
- Clinical users should apply the electrode belt at ICS 4–5 (parasternal line).
- If an out-of-phase signal or and overshoot signal exist, its origin should be clarified. If this signal is caused by a juxta-diaphragmatic belt position, it should be eliminated by repositioning the belt.
- Data containing out-of-phase signals should not be used for the analysis of ventilation-related impedance changes.
- If existing analysis tools are applied (e.g., “auto-scaling”) and if users do not take resultant effects into account, their findings might be biased and drawn conclusions are potentially wrong.

## Abbreviations

∆Z: Impedance change
CT: Computed tomography
EELV: End-expiratory lung volume
EIT: Electrical impedance tomography
ICS: Intercostal space
L: Layer
PEEP: Positive end-expiratory pressure
ROI: Region of interest
SAPS II: Simplified Acute Physiology Score II
TV/VT: Tidal impedance variation/tidal volume
VT: Tidal volume
